# Lower Incidence of End-Stage Renal Disease but Suboptimal Pre-Dialysis Renal Care in Schizophrenia: A 14-Year Nationwide Cohort Study

**DOI:** 10.1371/journal.pone.0140510

**Published:** 2015-10-15

**Authors:** Yueh-Han Hsu, Jur-Shan Cheng, Wen-Chen Ouyang, Chen-Li Lin, Chi-Ting Huang, Chih-Cheng Hsu

**Affiliations:** 1 Department of Public Health and Department of Health Services Administration, China Medical University, Taichung City, Taiwan; 2 Division of Nephrology, Department of Internal Medicine, Ditmanson Medical Foundation Chia-Yi Christian Hospital, Chia-Yi City, Taiwan; 3 Department of Nursing, Min-Hwei College of Health Care Management, Tainan City, Taiwan; 4 Clinical Informatics and Medical Statistics Research Center, College of Medicine, Chang Gung University, Taoyuan City, Taiwan; 5 Department of Psychiatry, Changhua Christian Hospital and Changhua Christian Healthcare System, Changhua, Taiwan; 6 Lutung Christian Hospital, Changhua, Taiwan; 7 Department of Nursing, College of Medicine and Life Science, Chung Hwa University of Medical Technology, Tainan, Taiwan; 8 Department of Psychiatry, Kaohsiung Medicine University, Kaohsiung, Taiwan; 9 Taipei City Hospital Fuyou Branch; Taipei, Taiwan; 10 Institute of Population Health Sciences, National Health Research Institutes, Zhunan, Miaoli County, Taiwan; 11 Department of Health Services Administration, China Medical University, Taichung City, Taiwan; University of Michigan, UNITED STATES

## Abstract

Schizophrenia is closely associated with cardiovascular risk factors which are consequently attributable to the development of chronic kidney disease and end-stage renal disease (ESRD). However, no study has been conducted to examine ESRD-related epidemiology and quality of care before starting dialysis for patients with schizophrenia. By using nationwide health insurance databases, we identified 54,361 ESRD-free patients with schizophrenia and their age-/gender-matched subjects without schizophrenia for this retrospective cohort study (the schizophrenia cohort). We also identified a cohort of 1,244 adult dialysis patients with and without schizophrenia (1:3) to compare quality of renal care before dialysis and outcomes (the dialysis cohort). Cox proportional hazard models were used to estimate the hazard ratio (HR) for dialysis and death. Odds ratio (OR) derived from logistic regression models were used to delineate quality of pre-dialysis renal care. Compared to general population, patients with schizophrenia were less likely to develop ESRD (HR = 0.6; 95% CI 0.4–0.8), but had a higher risk for death (HR = 1.2; 95% CI, 1.1–1.3). Patients with schizophrenia at the pre-ESRD stage received suboptimal pre-dialysis renal care; for example, they were less likely to visit nephrologists (OR = 0.6; 95% CI, 0.4–0.8) and received fewer erythropoietin prescriptions (OR = 0.7; 95% CI, 0.6–0.9). But they had a higher risk of hospitalization in the first year after starting dialysis (OR = 1.4; 95% CI, 1.0–1.8, *P* < .05). Patients with schizophrenia undertaking dialysis had higher risk for mortality than the general ESRD patients. A closer collaboration between psychiatrists and nephrologists or internists to minimize the gaps in quality of general care is recommended.

## Introduction

Schizophrenia has been linked to many physical illnesses [1, 2]. Recent evidence has shown that schizophrenia is closely associated with hypertension [3], metabolic syndrome [4], obesity [5], type 2 diabetes [3, 6], and dyslipidemia [3, 5]. Significantly higher risks of cardiovascular events such as coronary heart disease, stroke and congestive heart failure were reported in patients with schizophrenia [7–9]. Because of the fact that schizophrenia and chronic kidney disease (CKD) / end-stage renal disease (ESRD) shared many common cardiovascular risk factors, it is reasonable to speculate that the prevalence of CKD/ESRD might be higher in patients with schizophrenia. A recent research reported 25% increased risk of CKD in patients with schizophrenia from a nationwide cohort study [10]. However, no study has investigated the risk of ESRD and the quality of pre-dialysis renal care in patients with schizophrenia. In an analysis of the relationship between type 2 diabetes mellitus and schizophrenia, Schoepf et al reported chronic renal failure to be an important disease contributing to death in patients with schizophrenia [11]. CKD/ESRD might be a hidden risk factor contributing to survival and healthcare outcome in schizophrenia. Our first aim was to investigate the incidence of ESRD and mortality in patients with schizophrenia.

People with mental disorders face unmet healthcare needs for both mental and physical care [12–14]. The physical healthcare of patients with severe mental illnesses is often neglected, and contributes to the noticeable disparity in health [12]. For patients with schizophrenia, the therapeutic gap in untreated somatic diseases across the world has been estimated at 32.2% by the World Health Organization [15]. Nasrallah et al reported that, in schizophrenia, the rates of non-treatment for diabetes, hypertension and hyperlipidemia were 30.2%, 62.4% and 88.0% respectively [16]. The conditions of renal care of patients with schizophrenia were unknown.

With regard to CKD/ESRD patients, especially for those in the late stage, the quality of pre-dialysis renal care is a crucial determinant of health outcomes. Indices of good quality for pre-dialysis renal care include referral to nephrologists, erythropoietin (EPO) prescription, preparation for vascular access, and planned dialysis initiation; better pre-dialysis renal care also contributes to a lower risk of hospitalization and death [17–22]. Therefore, minimizing disparities in pre-dialysis renal care is an important issue for dialysis patients with schizophrenia. So far, no relevant research has investigated this topic; our second aim was to assess the quality of pre-dialysis renal care and prognosis in dialysis-dependent ESRD patients with schizophrenia.

## Materials and Methods

### Data Sources

The mandatory National Health Insurance (NHI) program in Taiwan started in 1995. In 2008, the NHI program covered over 99% of the population, and had signed healthcare contracts with more than 92% of healthcare organizations in Taiwan [23]. The data analyzed in this study were retrieved from the Taiwan NHI Research Database (NHIRD), which is released by the NHI Administration and maintained by the Taiwan National Health Research Institutes (NHRI). The Longitudinal Health Insurance Database for the year 2000 (LHID2000), one of the data components in NHIRD, is comprised of the registration and claims data of one million individuals who have been randomly selected from the 2000 Registry of Beneficiaries of the NHI program, and has proven to be representative of all NHI enrollees in terms of age and gender distribution [24].

The Registry of Catastrophic Illness Patients is another dataset of the NHIRD, containing the details of patients with all catastrophic illnesses defined by the Ministry of Health and Welfare. There has been a rigorous process of clinical review and evaluation before certifying applicants into the registry of catastrophic illness to assure the accuracy of the diagnoses. The catastrophic injuries/illnesses in Taiwan included 31 categories of major illnesses (for example: cancer, end-stage renal disease, hemophilia, and so on), with which patients are exempt from co-payment and may thus avoid financial hardship [25]. Schizophrenia (ICD-9-CM code: 295) and chronic dialysis-dependent ESRD (ICD-9-CM code: 585) are both listed as catastrophic illnesses. Therefore, the diagnoses of the selected study subjects (those with schizophrenia or chronic dialysis-dependent ESRD) had been validated through the process of strict administrative review as described in other studies [26–28].

All personal identification information was encrypted before the release of the research database to protect patient privacy. This study was granted ethical approval by the Institutional Review Board of the NHRI, Taiwan.

### Study subjects

**Fig 1:**
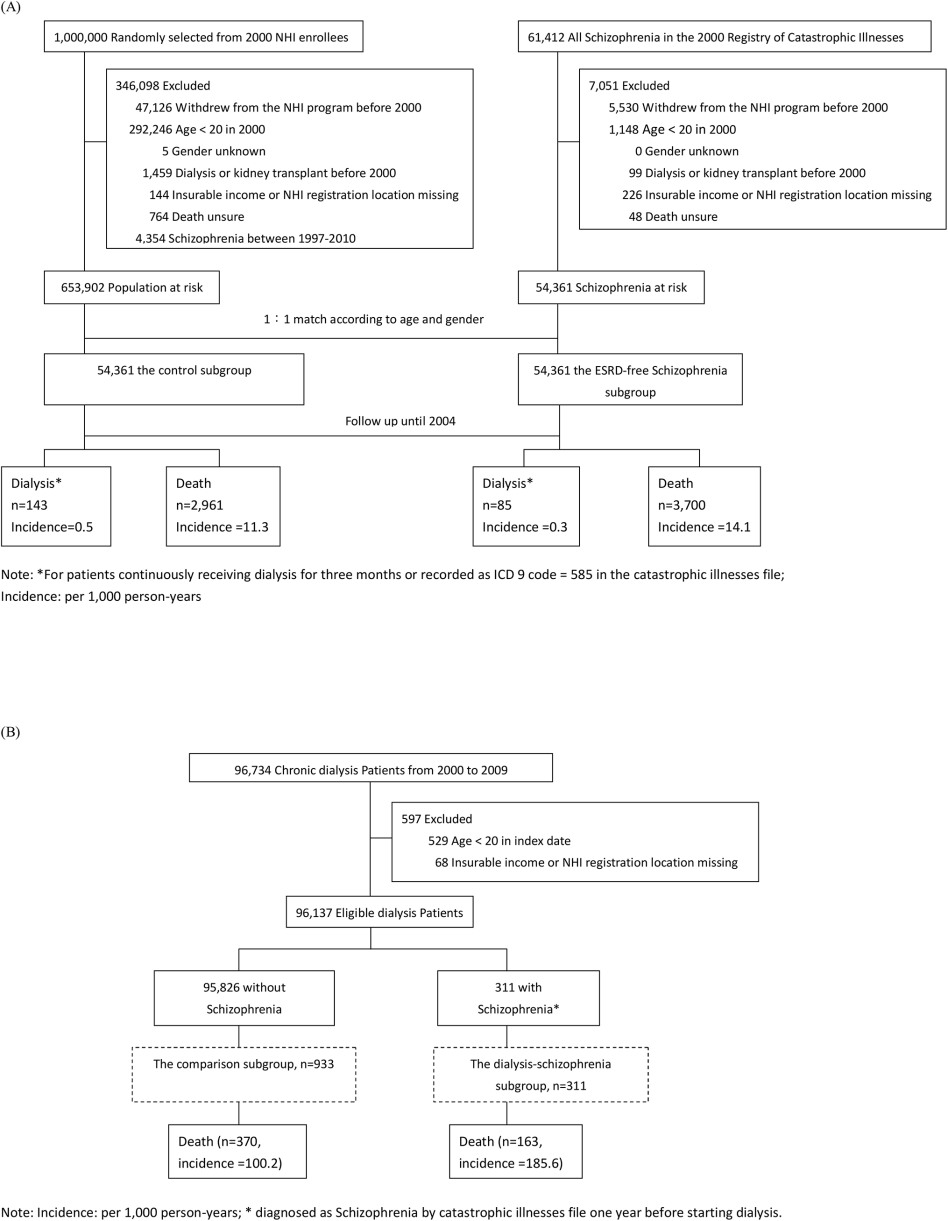
(A) The study flow chart of the Schizophrenia Cohort; (B) The study flow chart of the Dialysis Cohort.

To examine the risks of dialysis and death in people with schizophrenia, we assembled the schizophrenia cohort, consisting of ESRD-free schizophrenia and control subgroups. The ESRD-free schizophrenia subgroup included all ESRD-free NHI adult enrollees (age ≥ 20) who received a catastrophic illness certificate of schizophrenia before 2000; the exclusion criteria for the ESRD-free schizophrenia subgroup were: withdrawal from the NHI program before 2000, incomplete demographic or survival data, or on regular dialysis or kidney transplantation before 2000 (Fig 1A).

The control subgroup in the schizophrenia cohort included 1:1 age- and gender-matched subjects who were randomly selected from LHID2000, after exclusion of those with a diagnosis of schizophrenia between 1997 and 2010, or the conditions as listed in the exclusion criteria for the schizophrenia subgroup.

To compare the quality of pre-dialysis renal care and dialysis outcomes between dialysis patients with and without schizophrenia, we assembled a second study cohort from individuals holding a catastrophic illness certificate of chronic dialysis-dependent ESRD: the dialysis cohort, within which individuals with schizophrenia (the dialysis-schizophrenia subgroup) and without schizophrenia (the comparison subgroup) were compared. The dialysis-schizophrenia subgroup was those who started dialysis treatment in 2000–2009 and held a catastrophic illness certificate of schizophrenia before the dialysis commencement. The comparison subgroup was formed by selecting the dialysis patients with 1:3 age- and gender-matched to their dialysis-schizophrenia counterparts. (Fig 1B). The detailed study flow chart was shown in Fig 1.

### Definition of Variables

Utilization of medical services was determined from the NHI reimbursement claims, including all services and medicines covered by the NHI program. The initiation of chronic dialysis (index date) was defined as the date of starting dialysis for at least three consecutive months or the approval date of the catastrophic illness certificate for ESRD, whichever came first, to assure actual need and receipt of dialysis. Modes of dialysis included hemodialysis (HD) and peritoneal dialysis (PD).

Indicators of pre-dialysis renal care consisted of: (1) nephrologists visits six months prior to the initiation of dialysis; (2) EPO prescription six months before initiating dialysis; (3) for HD patients, vascular access prepared at least two weeks before starting HD; (4) initiation of dialysis through regular ambulatory clinics rather than via emergency room or admission for HD patients.

The outcomes after starting dialysis included: (1) hospitalization in the first year after dialysis; (2) death after dialysis.

### Statistical Analysis

The incidence rates for dialysis and death in two subgroups of the schizophrenia cohort were measured by the number of cases per 1,000 person-years. The person-years were calculated as the time from the entry date (January 1^st^, 2000) to the date of the event, death, or the end of follow-up (December 31^st^, 2004), whichever came first. Cox proportional hazards models were used to estimate adjusted hazard ratios for dialysis and death, adjusted for age and gender, NHI registration location (city, township, or rural area), insurable income level (poor, low income, middle income, or high income) [29], the Charlson comorbidity index (CCI) score (0, 1–2, >2) [30], and comorbid diabetes and hypertension.

To determine how schizophrenia affected pre-dialysis care and the risk of hospitalization in the first year after dialysis, conditional logistic models were adopted, adjusted for age, gender, NHI registration location, insurable income level, CCI score, and comorbid diabetes. Hospitalization in the year prior to starting dialysis was also adjusted when the risk of hospitalization after dialysis was evaluated.

Kaplan-Meier survival analysis was used to compare longitudinal changes in all-cause mortality between the 2 subgroups of the dialysis cohort. Cox proportional hazards analyses were adopted to compare all-cause mortality in the dialysis cohort, with respect to the status of schizophrenia (yes or no), diverse subgroups by age (20–49, 50–69, ≥ 70), gender (male, female), comorbid diabetes (yes, no), CCI score (≤2, >2), nephrologists visits within six months prior to the initiation of dialysis (yes, no), and years of initiation of dialysis (2000–2004, 2005–2009).

SAS software version 9.1 (SAS Institute, Cary, NC) was used to perform the analyses. For all difference estimates, we calculated 95% confidence intervals (95% CIs). P values < 0.05 were considered to be statistically significant.

## Results

### Risk of dialysis and death in the schizophrenia cohort

In the Registry of Catastrophic Illnesses Patients, there were 54,361 patients with schizophrenia in 2000 and they made up the ESRD-free schizophrenia subgroup of the schizophrenia cohort. From LHID2000, 54,361 subjects with 1:1 age and gender matched to the ESRD-free schizophrenia subgroup were selected as the control subgroup of the schizophrenia cohort. As shown in Table 1, more schizophrenic patients seemed to live in rural areas, had lower incomes, higher CCI scores, and higher rates of co-morbidity for diabetes and hypertension.

**Table 1:** Demographics of study subjects in 2 different cohorts with and without schizophrenia. CCI: Charlson Comorbidity Index, DM: diabetes mellitus, EPO: erythropoietin, NHI: National Health Insurance.

	Schizophrenia Cohort					Dialysis Cohort				
	Control subgroup (n = 54361)		ESRD-free schizophrenia subgroup (n = 54361)		*P* value	Comparison subgroup (n = 933)		Dialysis-schizophrenia subgroup (n = 311)		*P* value
Age, years ^a^ (%)					1.0000					1.0000
Mean (SD)	41.1 (12.6)		41.1 (12.5)			55.0 (12.8)		54.9 (12.7)		
20–39	28423	(52.3)	28423	(52.3)		114	(12.2)	38	(12.2)	
40–49	14747	(27.1)	14747	(27.1)		240	(25.7)	80	(25.7)	
50–59	6344	(11.7)	6344	(11.7)		249	(26.7)	83	(26.7)	
60–69	3158	(5.8)	3158	(5.8)		207	(22.2)	69	(22.2)	
>70	1689	(3.1)	1689	(3.1)		123	(13.2)	41	(13.2)	
Gender (%)					1.0000					1.0000
Male	29804	(54.8)	29804	(54.8)		393	(42.1)	131	(42.1)	
Female	24557	(45.2)	24557	(45.2)		540	(57.9)	180	(57.9)	
NHI registration location (%)					<0.0001					0.2675
City	16747	(30.8)	14039	(25.8)		235	(25.2)	76	(24.4)	
Township	15943	(29.3)	15509	(28.5)		318	(34.1)	93	(29.9)	
Rural area	21671	(39.9)	24813	(45.7)		380	(40.7)	142	(45.7)	
Income					<0.0001					<0.0001
Poor	185	(0.3)	5519	(10.1)		13	(1.4)	44	(14.1)	
Low income	7582	(14.0)	23787	(43.8)		195	(20.9)	116	(37.3)	
Middle income	43635	(80.3)	24348	(44.8)		676	(72.4)	138	(44.4)	
High income	2959	(5.4)	707	(1.3)		49	(5.3)	13	(4.2)	
CCI score					<0.0001					0.0382
0	47046	(86.5)	45841	(84.3)		110	(11.8)	54	(17.4)	
1–2	5841	(10.8)	7007	(12.9)		259	(27.8)	77	(24.8)	
>2	1474	(2.7)	1513	(2.8)		564	(60.5)	180	(57.9)	
Comorbidity										
DM	1567	(2.9)	2328	(4.3)	<0.0001	453	(48.6)	139	(44.7)	0.2380
Hypertension	2430	(4.5)	2630	(4.8)	0.0040	517	(55.4)	166	(53.4)	0.5319
Renal care ^b^										
Nephrologist visit						724	(77.6)	205	(65.9)	<0.0001
EPO treatment						430	(46.1)	113	(36.3)	0.0027
Dialysis modality ^c^										0.0004
Peritoneal dialysis						76	(9.3)	7	(2.7)	
Hemodialysis						739	(90.7)	255	(97.3)	
Permanent vascular access created in advance ^c^ ^,^ ^d^						188	(25.4)	53	(20.8)	0.1347
First time dialysis setting										0.3333
Emergency						47	(5.8)	18	(6.9)	
Out-patient clinic						142	(17.4)	36	(13.7)	
Hospitalization						626	(76.8)	208	(79.4)	

^a^ Age to be initially followed for schizophrenia cohort and age of initiating regular dialysis cohort

^b^ Conditions investigated six months prior to initiating regular dialysis

^c^ for patients continuously receiving dialysis for three months immediately after the index date

^d^ for patients on hemodialysis, and created vascular access at least 2 weeks before initiating regular dialysis

After 5 years of follow-up, there were 2,961 deaths and 143 ESRD subjects who required dialysis identified in the control subgroup, and 3,700 deaths and 85 ESRD subjects who required dialysis in the ESRD-free schizophrenia subgroup (Table 2). The incidence of dialysis and death in the control subgroup was 0.5 and 11.3 events per thousand person-years, respectively; in the ESRD-free schizophrenia subgroup, the corresponding incidence was 0.3 and 14.1 per thousand person-years, respectively. Compared with the control subgroup in the multivariate Cox regression model, the ESRD-free schizophrenia subgroup had a lower risk for dialysis (HR = 0.6, 95% CI 0.5–0.8, *P* < .001), but a higher risk for death (HR = 1.2, 95% CI 1.2–1.3, *P* < .001).

**Table 2:** Relative risk for dialysis or death in the schizophrenia cohort. HR: hazard ratio.

		Dialysis			Death		
Subgroup	N	n	Incidence^a^	HR^b^	n	Incidence^a^	HR^b^
The control ^c^	54361	143	0.5	1.0	2961	11.3	1.0
The ESRD-free schizophrenia^c^	54361	85	0.3	0.60 (0.45–0.81) **	3700	14.1	1.23 (1.17–1.30) **

^a^ Incidence per 1,000 person-years

^b^ Model is adjusted for age, gender, CCI score, comorbidity (DM, and hypertension), NHI registration location, and income

^c^ Patients were followed from 2000.01.01, until starting dialysis, death or end of the follow-up (2004.12.31), whichever came first

**P < 0.001.

### Pre-dialysis renal care and dialysis outcomes in the dialysis cohort

In the dialysis cohort, the group of dialysis-dependent ESRD patients, 311 with schizophrenia (the dialysis-schizophrenia subgroup) and 933 age and gender-matched non-schizophrenic subjects (the comparison subgroup) were followed through until the end of 2010. As shown in Table 1, patients in the dialysis-schizophrenia subgroup seemed to have lower incomes, higher CCI scores, lower chances of seeing nephrologists or receiving EPO prescription before dialysis, and were more likely to receive HD as the dialysis modality.

Compared to the comparison subgroup, the dialysis-schizophrenia subgroup had a lower chance of seeing nephrologists (OR = 0.6, 95% CI 0.4–0.8, *P* < .001), a lower likelihood of receiving EPO prescription (OR = 0.7, 95% CI 0.6–0.9, *P* < .05) within 6 months before starting dialysis, and a higher risk of hospital admission in the first year after starting dialysis (OR = 1.4, 95% CI 1.0–1.8, *P* < .05). (Table 3)

**Table 3:** All-cause mortality of the dialysis cohort stratified by different characteristics. CCI: Charlson Comorbidity Index, DM: diabetes mellitus, HR: hazard ratio, Nephrologist visit: in six months prior to dialysis initiation.

Variable	N	Death	Person-year	Mortality^a^	95% CI	*P* value	HR ^b^	95% CI	
**Overall**									
Comparison subgroup	933	370	3693.11	100.2	90–111	-	1.0	-	-
Dialysis-schizo subgroup	311	163	878.22	185.6	159–216	<0.0001	1.84	1.50	2.27
**Age 20–49**									
Comparison subgroup	354	93	1641.87	56.6	46–69	-	1.0	-	-
Dialysis-schizo subgroup	118	43	452.54	95.0	70–127	0.0329	1.57	1.04	2.39
**Age 50–69**									
Comparison subgroup	456	193	1775.13	108.7	94–125	-	1.0	-	-
Dialysis-schizo subgroup	152	84	379.83	221.2	178–272	<0.0001	1.86	1.39	2.49
**Age ≧70**									
Comparison subgroup	123	84	276.11	304.2	244–375	-	1.0	-	-
Dialysis-schizo subgroup	41	36	45.85	785.2	558–1075	0.0002	2.48	1.55	3.98
**Male**									
Comparison subgroup	393	146	1490.02	98.0	83–115	-	1.0	-	-
Dialysis-schizo subgroup	131	66	356.43	185.2	144–234	0.0003	1.97	1.36	2.84
**Female**									
Comparison subgroup	540	224	2203.09	101.7	87–116	-	1.0	-	-
Dialysis-schizo subgroup	180	97	521.80	185.9	152–226	<0.0001	1.76	1.36	2.29
**DM (yes)**									
Comparison subgroup	453	224	1500.95	149.2	131–170	-	1.0	-	-
Dialysis-schizo subgroup	139	85	291.64	291.5	234–359	<0.0001	1.88	1.42	2.48
**DM (no)**									
Comparison subgroup	480	146	2192.16	66.6	56–78	-	1.0	-	-
Dialysis-schizo subgroup	172	78	586.58	133.0	106–165	0.0002	1.84	1.33	2.55
**CCI score≦2**									
Comparison subgroup	369	112	1721.11	65.1	54–78	-	1.0	-	-
Dialysis-schizo subgroup	131	58	418.12	138.7	106–178	0.0155	1.61	1.10	2.38
**CCI score>2**									
Comparison subgroup	564	258	1972.01	130.8	116–148	-	1.0	-	-
Dialysis-schizo subgroup	180	105	460.10	228.2	188–275	<0.0001	1.84	1.43	2.38
**Nephrologist visit (yes)**									
Comparison subgroup	724	269	2903.50	92.7	82–104	-	1.0	-	-
Dialysis-schizo subgroup	205	112	550.04	203.6	168–244	<0.0001	2.19	1.72	2.80
**Nephrologist visit (no)**									
Comparison subgroup	209	101	789.61	127.9	105–155	-	1.0	-	-
Dialysis-schizo subgroup	106	51	328.19	155.4	117–203	0.1410	1.36	0.90	2.05
**Year 2000–2004**									
Comparison subgroup	457	231	2378.37	97.1	85–110	-	1.0	-	-
Dialysis-schizo subgroup	103	75	404.33	185.5	147–231	<0.0001	1.99	1.47	2.70
**Year 2005–2009**									
Comparison subgroup	476	139	1314.75	105.7	89–124	-	1.0	-	-
Dialysis-schizo subgroup	208	88	473.90	185.7	150–228	<0.0001	1.80	1.34	2.40

^a^ Mortality: number per 1,000 person-years

^b^ Model is adjusted for age, gender, NHI registration location, income, CCI score, DM, nephrologists visit and year

The cumulative survival analysis revealed that the dialysis-schizophrenia subgroup had a significantly lower survival rate (log rank test: *P* < .0001; detailed in the S1 Fig). The incidence of death for dialysis patients with and without schizophrenia was 185.6 and 100.2 events per thousand person-years, respectively (Table 4).

**Table 4:** Quality of pre-dialysis renal care in the dialysis cohort. CCI: Charlson Comorbidity Index, EPO: erythropoietin, OR: odds ratio.

Care indicator	Comparison subgroup (n = 933)			Dialysis-schizophrenia subgroup (n = 311)		
	%	OR	95%CI	%	OR	95%CI
Nephrologist visits ^a^ ^,^ ^e^	77.6	1.0	-	65.9	0.58	(0.42–0.79) **
EPO treatment ^b^ ^,^ ^e^	46.1	1.0	-	36.3	0.70	(0.53–0.93) *
Permanent vascular access created in advance ^c^ ^,^ ^e^	25.4	1.0	-	20.8	0.78	(0.55–1.11)
First dialysis as outpatient ^d^ ^,^ ^e^	18.7	1.0	-	14.5	0.74	(0.50–1.09)
Hospitalization in the first year after dialysis ^f^	46.4	1.0	-	53.4	1.36	(1.04–1.78) *

^a^ at least one visit six months prior to initiating regular dialysis

^b^ at least one record of receiving EPO treatment six months prior to initiating regular dialysis

^c^ for hemodialysis patients only

^d^ for patients continuously receiving dialysis for three months immediately after the index date

^e^ Conditional logistic regression model, adjusted for age, gender, area, income, CCI score, comorbid DM and HT

^f^ Conditional logistic regression model, adjusted for age, gender, area, income, CCI score, DM, HT, and hospitalization in one year before dialysis

*P < 0.05.

**P < 0.01.

The multivariate Cox proportional hazards model as shown in Table 4 revealed that dialysis–schizophrenia subgroup generally had an 84% higher risk (HR = 1.84, 95% CI 1.50–2.27, *P* < .0001) for mortality compared to the comparison subgroup. Excess mortality was also observed in most of the subgroup analyses, irrespective of differences in gender, age, severity of comorbid conditions, and the year of commencement of dialysis.

## Discussion

In this nationwide cohort study, we found that patients with schizophrenia had a 40% lower risk for dialysis, yet a 23% higher risk of death than the age- and gender-matched non-schizophrenic individuals. Dialysis patients with schizophrenia received inferior pre-dialysis renal care as evidenced by less access to nephrologist visits and less EPO prescription. Once they became dialysis-dependent, patients with schizophrenia had a 1.84 times higher risk of mortality regardless of their age, gender, disease severity or diabetic status, and had a higher risk of being admitted to hospital in the first year of dialysis. In brief, patients with schizophrenia had a lower risk for ESRD, received suboptimal pre-dialysis renal care and incurred a higher risk of mortality regardless of being dialysis-dependent or not.

Our data showed that patients with schizophrenia had a 40% lower risk of developing ESRD requiring dialysis, but a 20% higher risk of death compared to their non-schizophrenic counterparts. There are several possible reasons for this: first, patients with schizophrenia are reported to have two to three folds increase in mortality rate over the general population [31]; this supports our findings, as such a competing risk of death might explain the reduced risk for ESRD. Second, as shown in Table 1, patients with schizophrenia and ESRD have a 40% lower chance of visiting nephrologists and a 30% lower chance of receiving EPO treatment within six months prior to starting dialysis. Lower access and adherence to supportive treatment were seen as potential factors in the increased mortality in patients with severe mental illnesses [32].

This research provides the first evidence addressing the unmet need for pre-dialysis renal care in the schizophrenic population. Suboptimal pre-dialysis renal care, less access to nephrologists’ visits, and fewer chances for EPO prescription in patients with schizophrenia coincided with their higher risk for hospital admission and death after dialysis. These findings are consistent with previous studies [17–19, 21, 22]. In fact, the neglect of physical well-being in patients with severe mental illnesses, including schizophrenia, is often attributable to an egregious disparity in health [12, 13]. Nasrallah et al reported a high proportion of under-diagnosis and under-treatment of hypertension, dyslipidemia and diabetes in schizophrenia [16]. Individuals with severe mental illnesses including schizophrenia who have had an acute myocardial infarction (AMI) appeared to be less likely to receive evidence-based treatments such as coronary artery bypass graft surgery (CABG) or percutaneous transluminal coronary angiography (PTCA) [33]. Kurdyak et al reported that patients with schizophrenia and AMI had a 1.56 fold higher mortality rate, yet 52% and 47% lower chances of receiving a cardiac procedure or seeing a cardiologist [33]. People with schizophrenia also have less access to health examinations or screening check-ups. Under the NHI program in Taiwan, the utilization rate of health examinations by the severely mentally ill was 22.32% [34], while the rate in the general population was 33.3 to 46.8% [35]. Healthcare disparity for patients with schizophrenia seems to be multi-dimensional, and the medical community should collectively take the initiatives to improve it.

Our data revealed that the mortality rate in schizophrenia was higher than that in non-schizophrenia, including those without dialysis (the schizophrenia cohort) and the subjects with ESRD requiring dialysis (the dialysis cohort). Schizophrenic patients with ESRD also had independently higher mortality regardless of their age, gender, co-morbid conditions, and income. Potential explanations include: first, patients with schizophrenia have a higher prevalence of cardio-metabolic risk factors including obesity, smoking tobacco, and diabetes [36, 37], which, in turn, increase CVD events [33]. Second, CVD remains the main cause of death in patients with schizophrenia, but those individuals are less likely to engage in secondary preventive strategies such as smoking cessation, exercise, body weight management or nutrition control [38]. Our data also revealed that more patients with schizophrenia had low incomes and lived in rural areas. This is consistent with the findings of Druss et al [39] that a large number of persons with serious mental illness were poor and uninsured, thus leading to poorer health. In addition, Torres-Gonzalez et al commented that inadequate implementation of clinical practice guidelines, uneven resource allocation and the stigma of seeing a psychiatrist contributed to the unmet needs in the management of schizophrenia [14]. We urge psychiatrists, general practitioners, internists, and nephrologists to be aware that the schizophrenic population has low access to health screening, inadequate pre-dialysis renal care, and higher mortality. Closer multidisciplinary collaboration to enhance health care access and the quality of renal care might help improving the situations.

There were several limitations to this research. First, this was a secondary dataset research based on different data files in which risk factors for ESRD (such as herbal therapy and intravenous drug use), and information about healthy life styles (avoiding smoking and alcoholism; regular exercise, healthy diets) were unavailable. The potential confounding effects associated with these factors becomes inevitable. Second, as the characteristics of NHIRD, we had no access to lab results, body mass index, and data regarding the drug-interactions among treatments for physical and mental disorders; a possible selection bias might compromise the present findings. Third, the identified co-morbid conditions [30] were based on the International Classification of Diseases codes (ICD-CM-9), which are less accurate than those obtained through standardized examinations and laboratory procedures. Since both ESRD requiring dialysis and schizophrenia are groups in catastrophic illnesses, that bias has been reduced to a minimum through the prudent certifying process. Finally, since the study subjects in this research were of Chinese ethnicity; the generalizability of our findings to other ethnic groups may be limited.

Patients with schizophrenia had a lower risk of ESRD requiring dialysis but a higher risk of death. Patients with schizophrenia in the late stage of CKD received suboptimal pre-dialysis renal care, including less access to nephrologists and fewer chances to receive EPO prescription. In the overall analysis, dialysis patients with schizophrenia had an 84% higher risk for mortality. Patients with schizophrenia also had significantly higher all-cause mortality regardless of age, gender, diabetic status, or different CCI scores. Closer collaboration between psychiatrists and nephrologists or internists to enhance attention to and treatment of CKD in individuals with schizophrenia is recommended.

## Supporting Information

S1 Fig The cumulative survival analysis of dialysis patients with and without schizophrenia. (TIF)
