# Supplementary figures and images for: Lower Incidence of End-Stage Renal Disease but Suboptimal Pre-Dialysis Renal Care in Schizophrenia: A 14-Year Nationwide Cohort Study
Source: PLoS One. 2015 Oct 15;10(10):e0140510. doi: 10.1371/journal.pone.0140510 (PMC4607300; doi:10.1371/journal.pone.0140510)

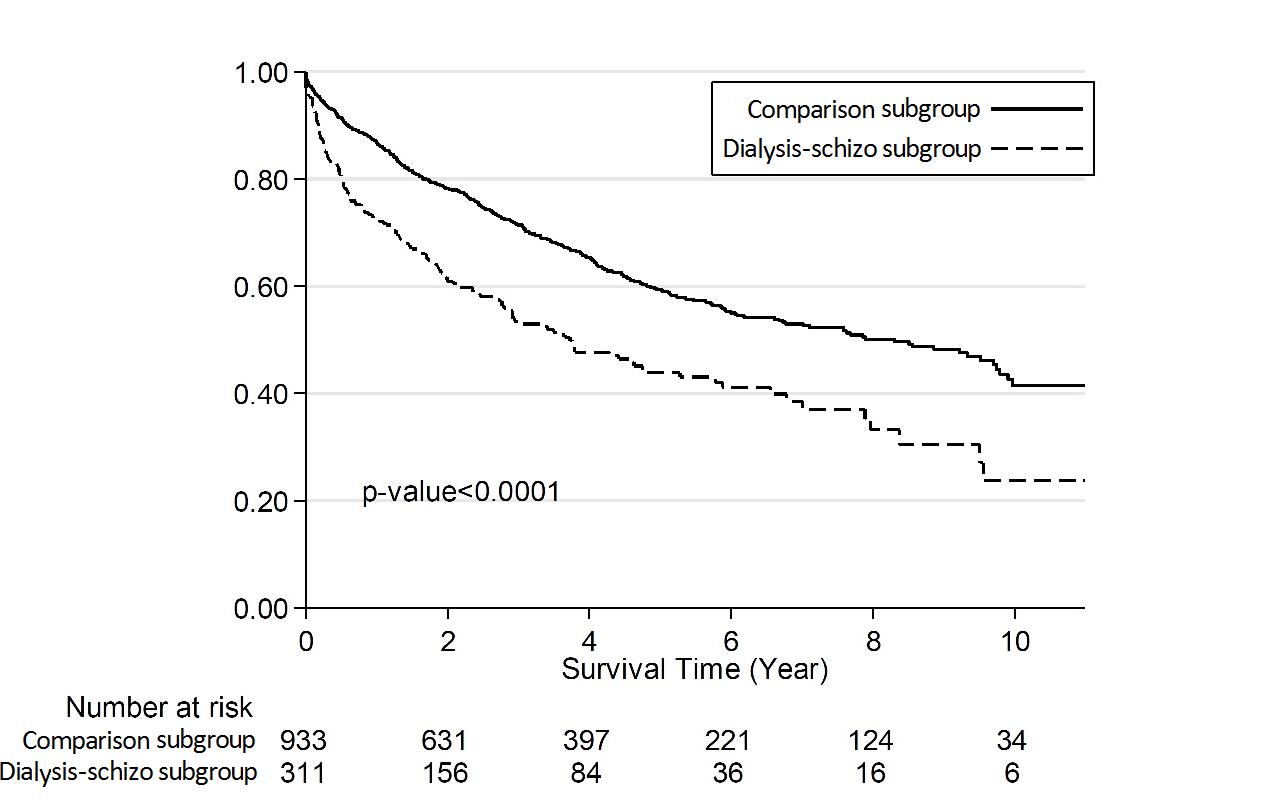

Supplement: S1 Fig — (TIF) [file pone.0140510.s001.tif]
